# Supplementary material for: Future climate change vulnerability of endemic island mammals
Source: Nat Commun. 2020 Oct 2;11:4943. doi: 10.1038/s41467-020-18740-x (PMC7532204; doi:10.1038/s41467-020-18740-x)
Supplement: Supplementary file 1 — Supplementary Information [file 41467_2020_18740_MOESM1_ESM.pdf]

Supplementary Information – Future climate change vulnerability of endemic island mammals – Leclerc et al.

Supplementary Information contains:

- Eleven Supplementary Figures:

Figure 1 Relationship between climate change vulnerability (RCP 6.0 – 2050 – basic standardization) and endemic species richness at archipelagos and islands level.
Figure 2 Correlation coefficients between standardization methods for adaptive capacity, exposure, sensitivity, and vulnerability for pools of insular endemic mammals face with future climate change (RCP 6.0, 2050).
Figure 3 Correlation coefficients between standardization methods and RCP scenarios for exposure and vulnerability for pools of insular endemic mammals face with future climate change (2050).
Figure 4 Islands-level robustness analyses of variables used for the different vulnerability components (RCP 6.0 – 2050 – basic standardization).
Figure 5 Archipelagos-level robustness analyses of variables used for the different vulnerability components (RCP 6.0 – 2050 – basic standardization).
Figure 6 Eigenvalues and explained variances of principal components, and PCA biplot of the four first principal components.
Figure 7 Relationships between the ecological characteristics and vulnerability to climate change at archipelagos scale.
Figure 8 Relationship between climate change vulnerability and climate change heterogeneity of islands, and its associated distribution density (RCP 6.0 – 2050 – basic standardization).
Figure 9 Spatial distributions of endemic mammal species richness studying within the 340 islands.
Figure 10 Correlation coefficients between variables of exposure, sensitivity and adaptive capacity.
Figure 11 Conceptual framework exemplifying the multicriteria method employed for the quantification of islands vulnerability.

- Three Supplementary Tables:

Table 1 List of variables characterizing the dimensions of the Principal Component Analysis.
Table 2 Number of islands within each archipelago.
Table 3 Measurement and source of variables characterizing the three vulnerability components.

- Supplementary Methods:

- **Procedure employed to select protected areas**

- Supplementary References

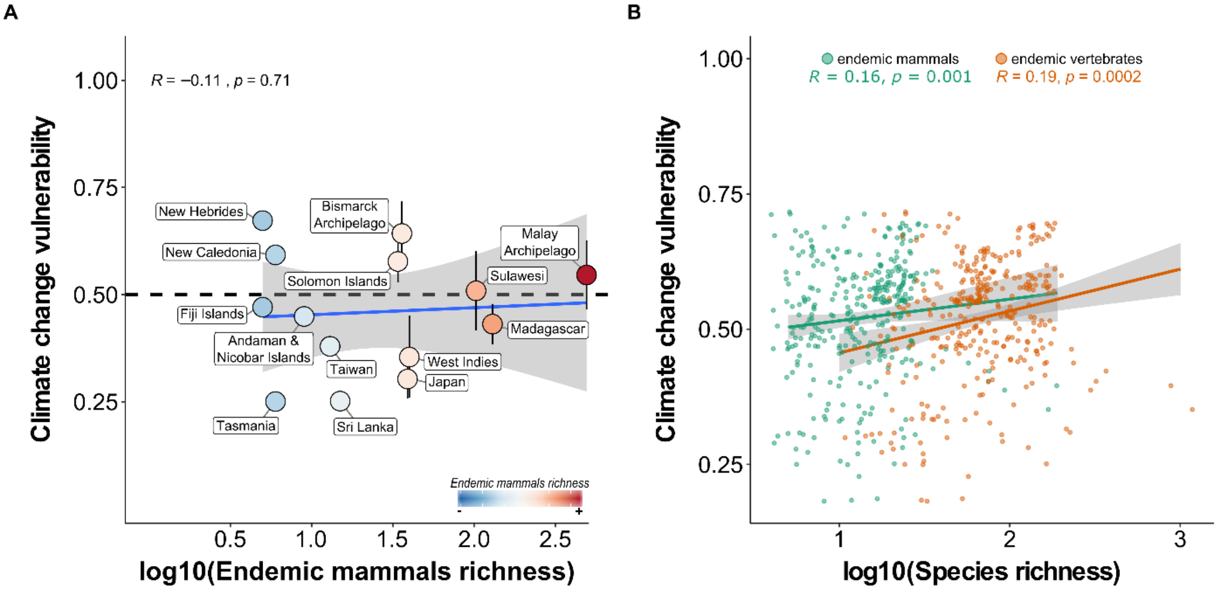

Supplementary Figure 1. Relationship between climate change vulnerability (RCP 6.0 – 2050 – basic standardization) and endemic species richness (log-transformation) at (A) archipelagos and (B) islands level. Spearman correlation coefficients and smooth curves based on a linear method with error bands representing a 95% confidence level are shown. In panel A, colour points represent mean values and associated error bars represent standard deviation values. Source data are provided as a Source Data file.

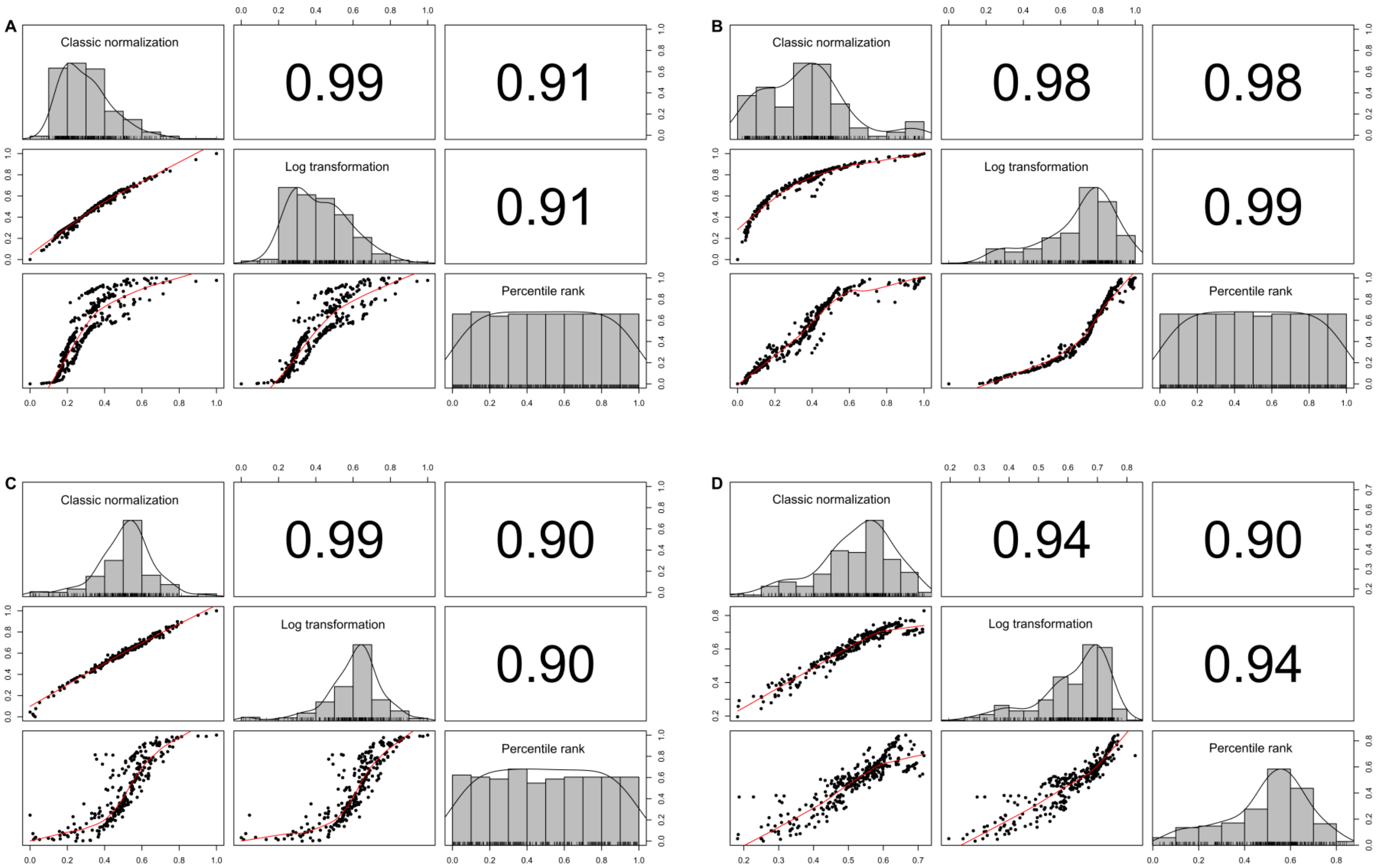

Supplementary Figure 2. Correlation coefficients (Spearman) between standardization methods (basic standardization, log transformation, and percentile rank) for (a) adaptive capacity, (b) exposure, (c) sensitivity, and (d) vulnerability for pools of insular endemic mammals face with future climate change (RCP 6.0, 2050). Source data are provided as a Source Data file.

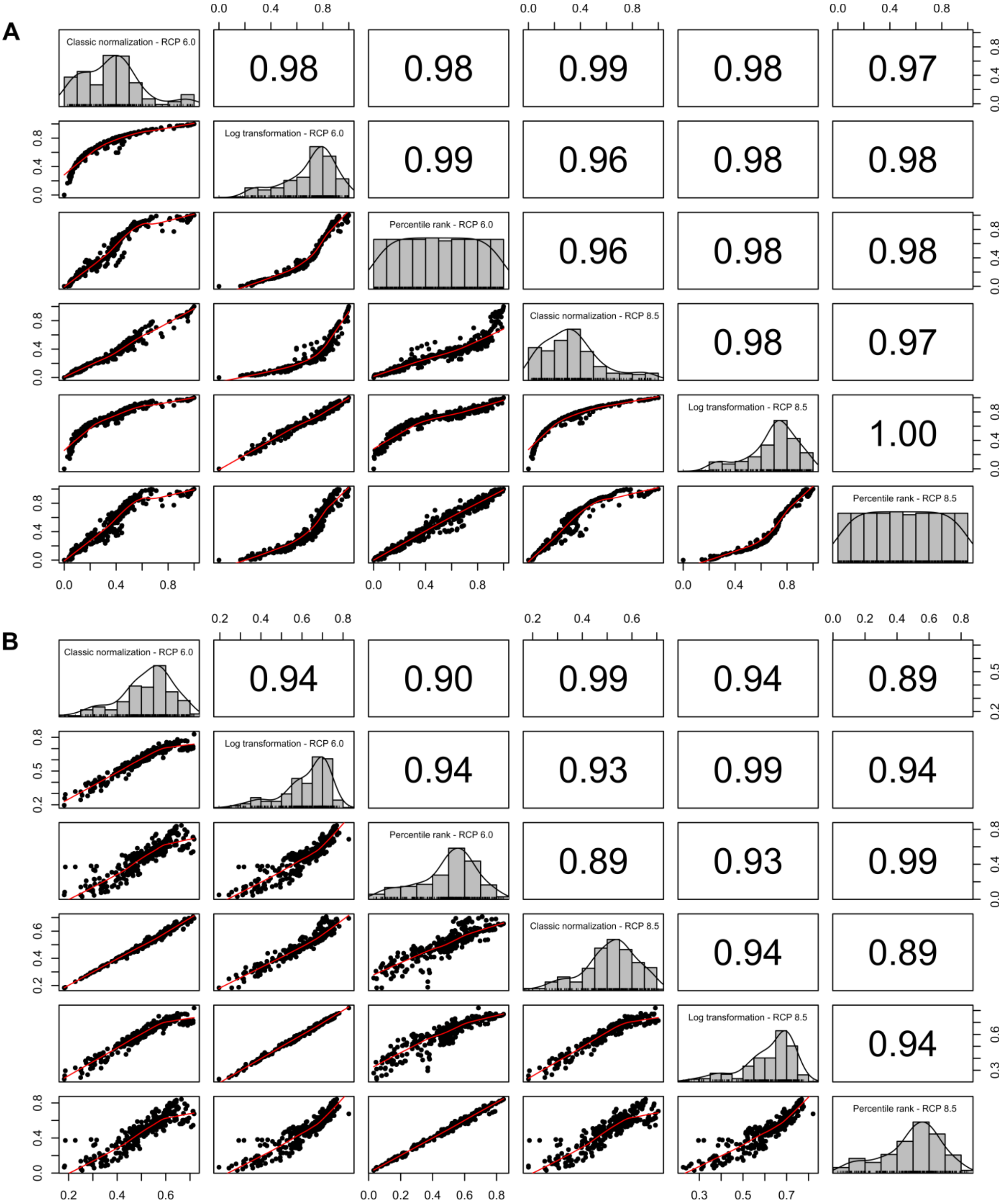

Supplementary Figure 3. Correlation coefficients (Spearman) between standardization methods (basic standardization, log transformation, and percentile rank) and RCP scenarios (6.0 and 8.5) for (a) exposure and (b) vulnerability for pools of insular endemic mammals face with future climate change (2050). Source data are provided as a Source Data file.

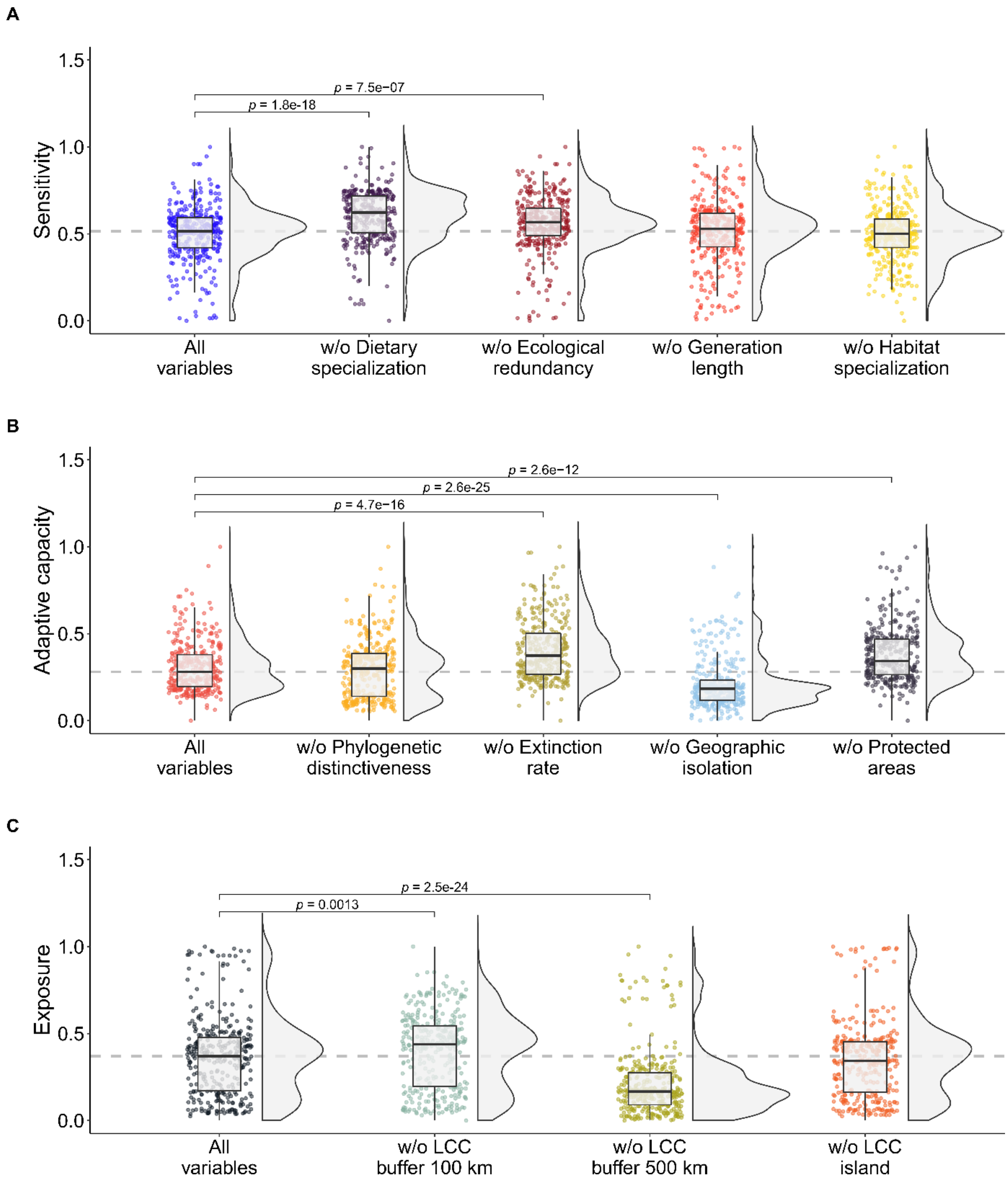

Supplementary Figure 4. Islands-level robustness analyses of variables used for the different vulnerability components: (a) sensitivity, (b) adaptive capacity, (c) exposure (RCP 6.0 – 2050 – basic standardization). "All variables" represent the observed values of islands obtained when all variables of a vulnerability component are considered. "w/o *variable name*" represents the values of islands obtained when the *variable* is not considered. Wilcoxon Mann-Whitney (two-sided) tests were applied to test of the difference between the distributions of "All variables" data and "w/o *variable name*" data. Only significant results are showed. Horizontal grey dashed lines represent median value of vulnerability component (sensitivity, adaptive capacity or exposure) when all variables are considered. Box plots indicate median (middle line), 25th, 75th percentile (box) and 5th and 95th percentile (whiskers). Source data are provided as a Source Data file.

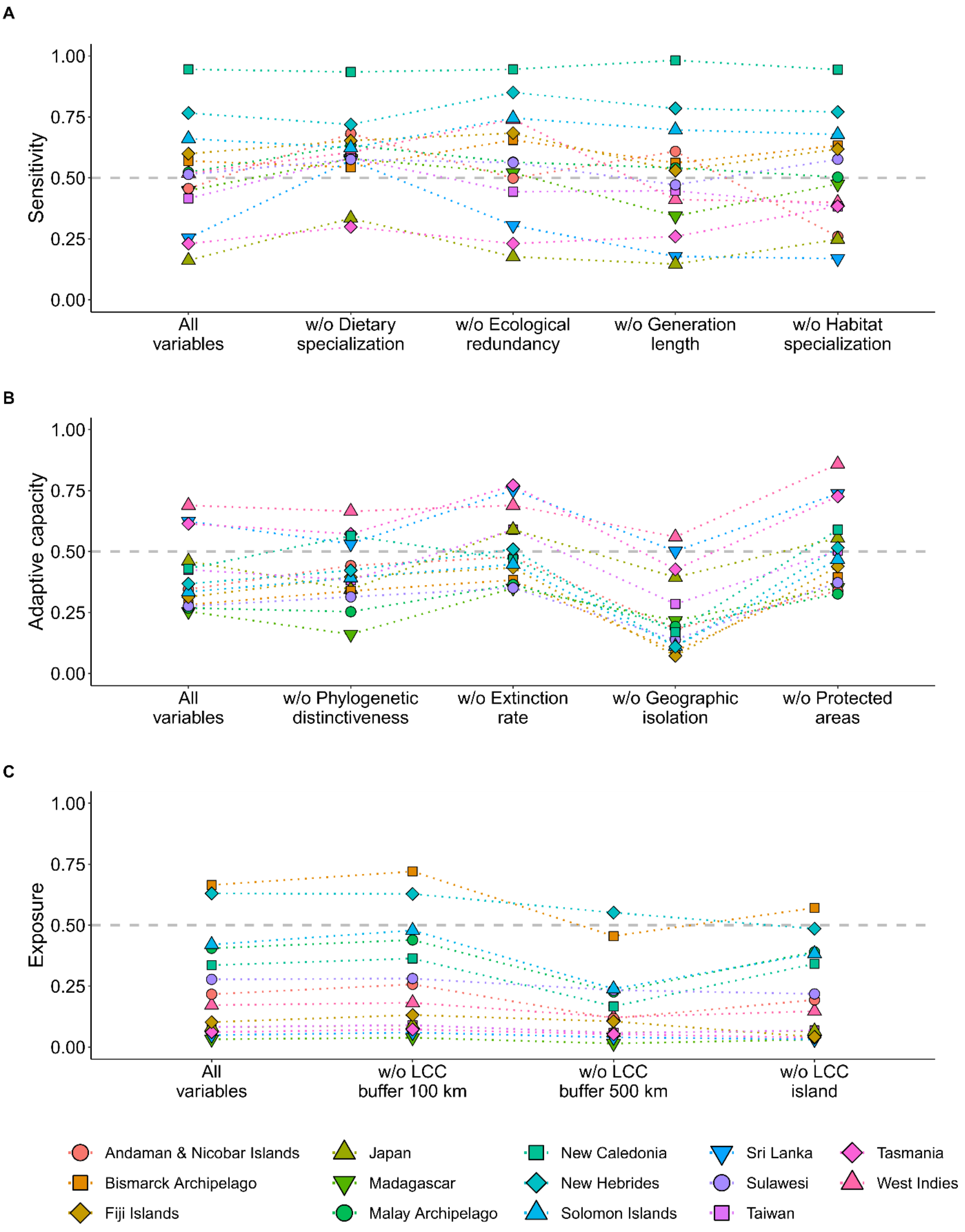

Supplementary Figure 5. Archipelagos-level robustness analyses of variables used for the different vulnerability components: (a) sensitivity, (b) adaptive capacity, (c) exposure (RCP 6.0 – 2050 – basic standardization). "All variables" represent the observed values of archipelagos obtained when all variables of a vulnerability component are considered. "w/o *variable name*" represents the values of archipelagos obtained when the *variable* is not considered. Horizontal grey dashed lines represent a 0.5 value of vulnerability component (sensitivity, adaptive capacity or exposure). Source data are provided as a Source Data file.

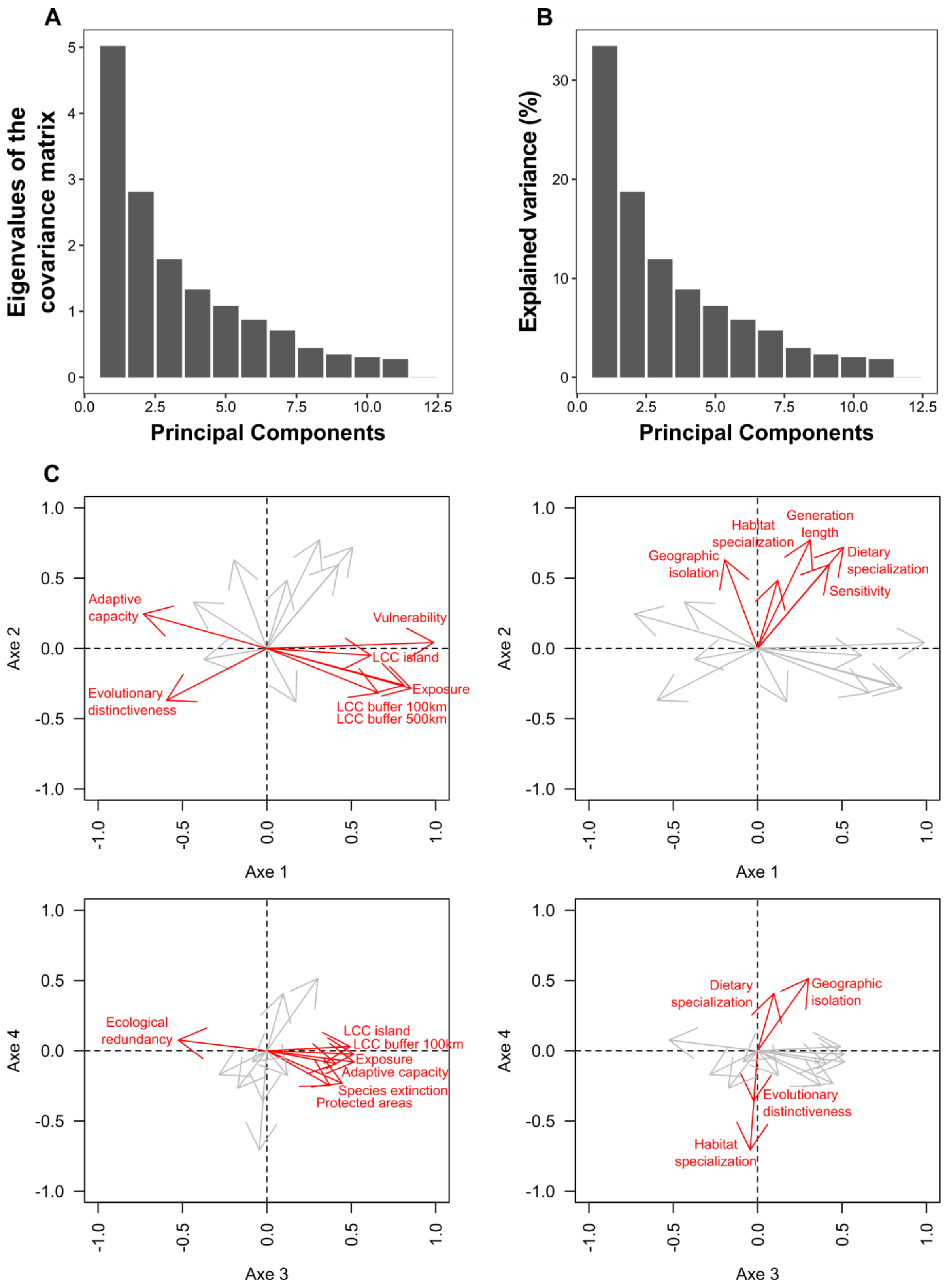

Supplementary Figure 6. (a) Eigenvalues and (b) explained variances of principal components, and (c) the PCA biplot of the four first principal components (RCP 6.0 – 2050 – basic standardization). Red variables indicate those which contributed at axis 1 (panel top left), 2 (panel top right), 3 (panel bottom left), and 4 (panel bottom right). Source data are provided as a Source Data file.

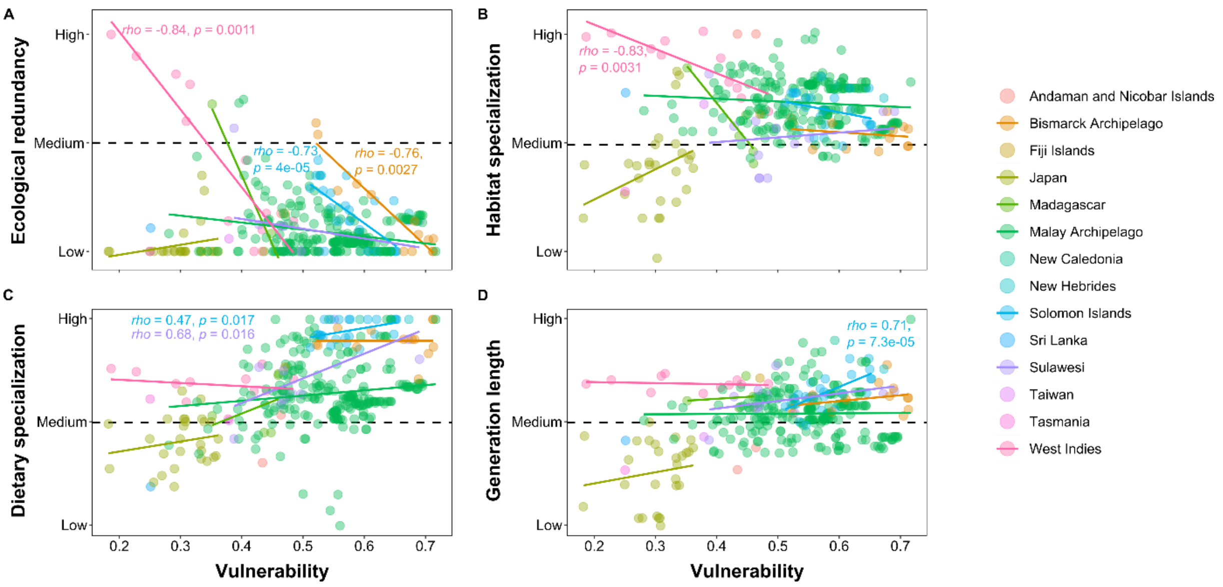

Supplementary Figure 7. Relationships between the ecological characteristics and vulnerability to climate change of insular endemic mammal pools (n = 340; RCP 6.0 – 2050 – basic standardization). Relationships between climate change vulnerability and (a) ecological redundancy, (b) habitat specialization, (c) dietary specialization, (d) generation length. Points are colored by archipelagos. Linear curves and significant Spearman correlation coefficients are shown. Source data are provided as a Source Data file.

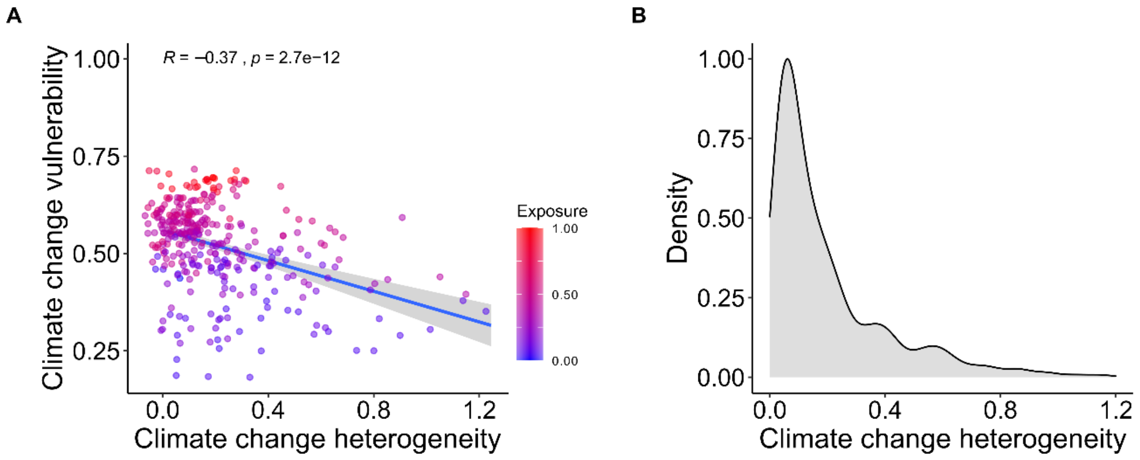

Supplementary Figure 8. (A) Relationship between climate change vulnerability (basic standardization) and climate change heterogeneity of islands (RCP 6.0 – 2050). (B) Distribution of climate change heterogeneity values of islands (RCP 6.0 – 2050). Climate change heterogeneity is the coefficient of variation (CV) of island local climate change (LCC) values ($CV_{island\ i} = \frac{\mathrm{sd}(LCC_{island\ i})}{\mathrm{mean}(LCC_{\ island\ i})}$) and expresses the relative variation of LCC within each island. Points are colored by climate change exposure values (RCP 6.0 – 2050 – basic standardization). Spearman correlation coefficient and smooth curve based on a linear method with error bands representing a 95% confidence level are shown. Source data are provided as a Source Data file.

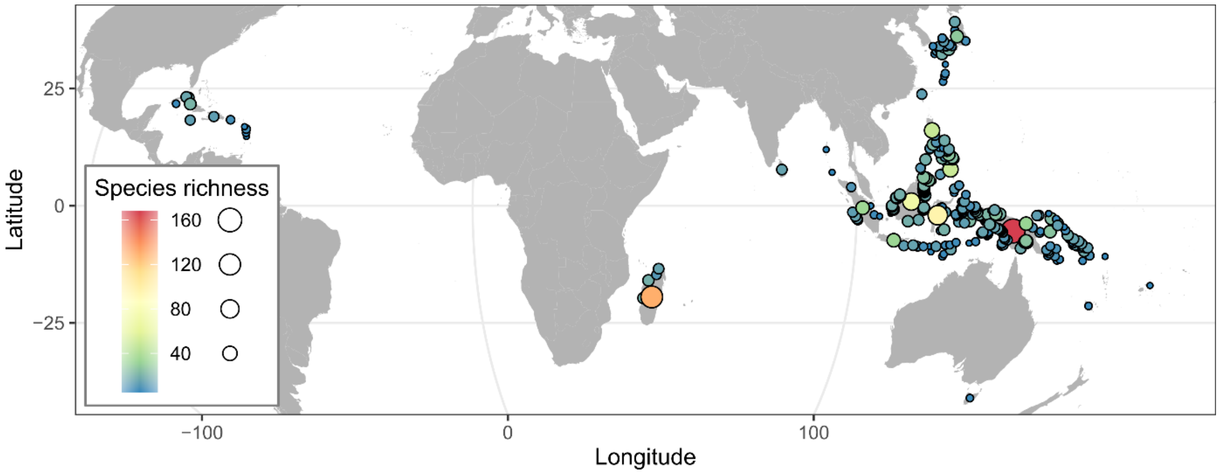

Supplementary Figure 9. Spatial distribution of endemic mammal species richness studying within the 340 islands. The number of endemic mammal species is shown for the mass centroid of the geographic island. Natural breaks classification, Mollweide projection. Source data are provided as a Source Data file.

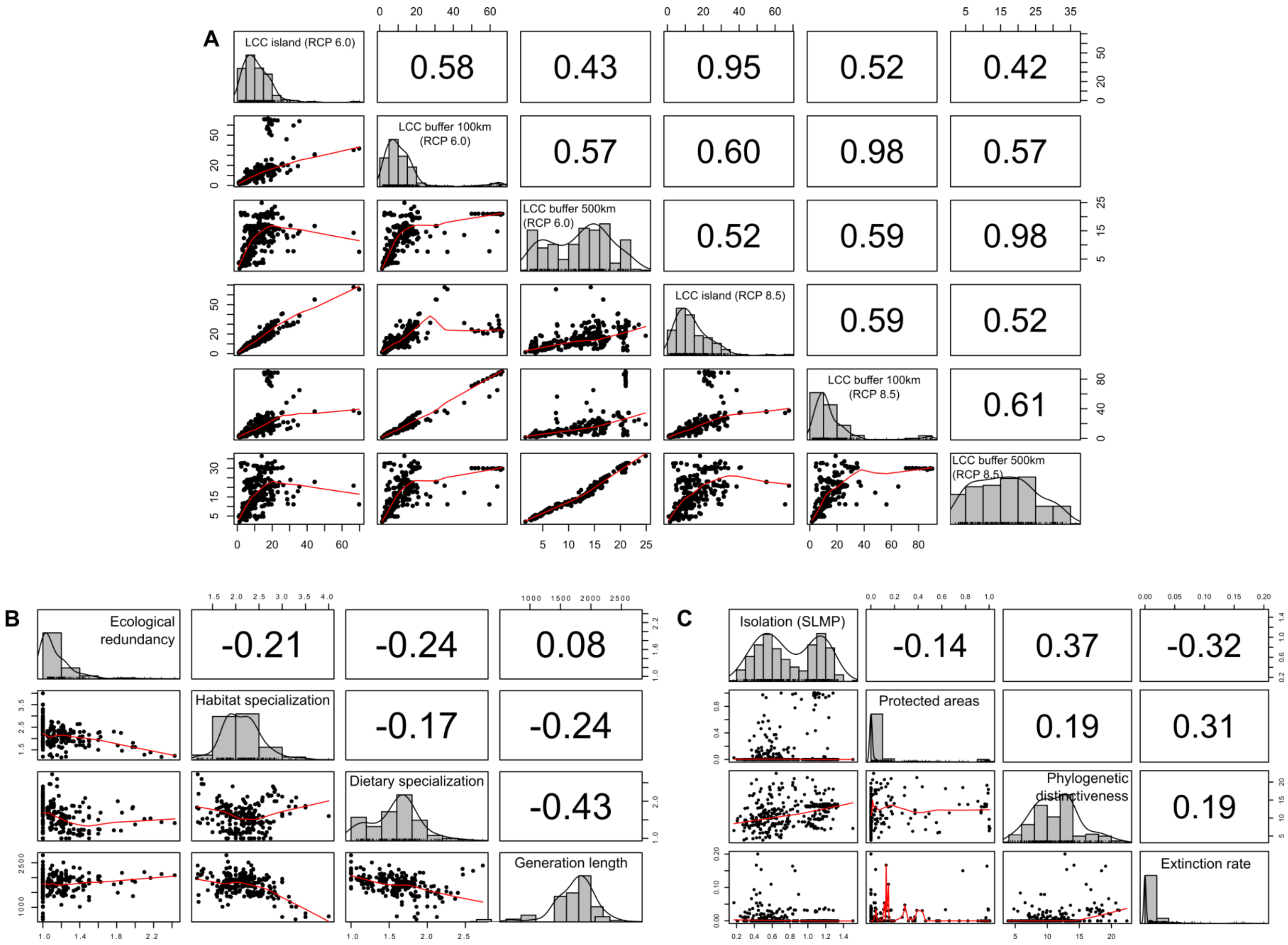

Supplementary Figure 10. Correlation coefficients (Spearman) between variables of (a) exposure, (b) sensitivity and (c) adaptive capacity. Source data are provided as a Source Data file.

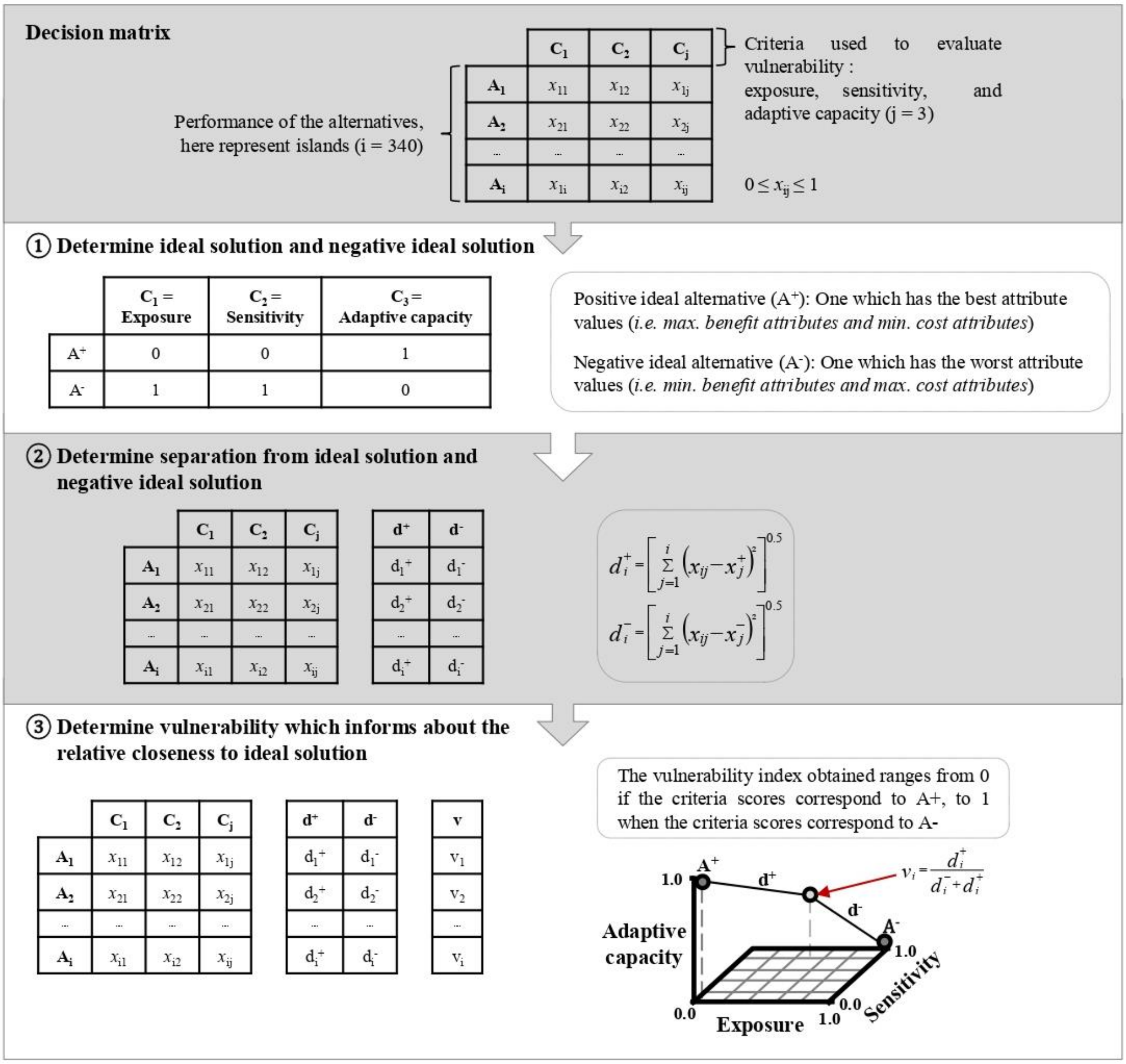

Decision matrix:

	C_1	C_2	C_j
A_1	x_{11}	x_{12}	x_{1j}
A_2	x_{21}	x_{22}	x_{2j}
...	...	...	...
A_i	x_{1i}	x_{i2}	x_{ij}

	C_1 = Exposure	C_2 = Sensitivity	C_3 = Adaptive capacity
A^+	0	0	1
A^-	1	1	0

	C_1	C_2	C_j	d^+	d^-	v
A_1	x_{11}	x_{12}	x_{1j}	d_1^+	d_1^-	v_1
A_2	x_{21}	x_{22}	x_{2j}	d_2^+	d_2^-	v_2
...	...	...	...	...	...	...
A_i	x_{i1}	x_{i2}	x_{ij}	d_i^+	d_i^-	v_i

Supplementary Figure 11. The conceptual framework exemplifying the multicriteria method employed for the quantification of islands vulnerability. Vulnerability is the result of exposure, sensitivity, and adaptive capacity. The light grey point represents an island to be evaluated. The vulnerability of the focal island (in light grey) is quantified as its relative distances to the positive ideal solution A^+ (here, minimum vulnerability = minimum exposure, minimum sensitivity and maximum adaptive capacity) and the negative ideal solution A^- (here, maximum vulnerability = maximum exposure, maximum sensitivity and minimum adaptive capacity). Vulnerability (v) is then numerically quantified as $v = d^+/(d^+ + d^-)$.

Supplementary Table 1. List of variables characterizing the dimensions of the Principal Component Analysis. Significantly correlated variables (based on pearson correlation) are shown for each dimension (p-value << 0.0001).

Variables	Dimension 1	Dimension 2	Dimension 3	Dimension 4
Adaptative capacity	-0.731	0.245	0.515	--
Dietary specialization	0.424	0.596	--	-0.407
Ecological redundancy	0.172	-0.380	-0.525	--
Phylogenetic distinctiveness	-0.594	-0.369	--	0.356
Exposure	0.855	-0.284	0.409	--
Generation length	0.311	0.771	-0.173	0.266
Geographic isolation	-0.195	0.630	0.301	-0.513
Habitat specialization	0.118	0.484	--	0.706
Local climate change within a 100 km buffer around each island	0.660	-0.317	0.513	--
Local climate change within a 500 km buffer around each island	0.811	-0.262	0.122	0.175
Local climate change within islands	0.616	--	0.492	--
Protected areas	-0.373	--	0.374	0.248
Sensitivity	0.509	0.719	-0.283	0.170
Extinction rate	-0.434	0.328	0.445	0.231
Vulnerability	0.988	--	--	--

Supplementary Table 2. Number of islands considered in our study within each archipelago.

Archipelago	Islands number
Andaman and Nicobar Islands	2
Bismarck Archipelago	13
Fiji Islands	1
Japan	27
Madagascar	5
Malay Archipelago	239
New Caledonia	1
New Hebrides	1
Solomon Islands	25
Sri Lanka	1
Sulawesi	12
Taiwan	1
Tasmania	1
West Indies	11

Supplementary Table 3. Measurement and source of variables characterizing the three vulnerability components (exposure, adaptive capacity, and sensitivity).

	**Measurement (source)**
Exposure	
Local climate change †<br>within each island<br>within a 100 km<br>within a 500 km	Standardized Euclidean Distance between 20th and 21st-century realizations for each gridpoint [1,2]
Sensitivity	
Habitat specialization *	Number of habitat used by each species [3]
Dietary specialization *	Number of modes of feeding by species [4]
Generation length *	Average age of parents of the species current cohort [5]
Ecological redundancy *	Species richness per functional group, i.e. that harbor unique combination of functional trait values [6]
Adaptive capacity	
Geographic isolation	Proportion of surrounding landmass around island perimeter [7]
Protected areas	Ratio of island area covered by protected areas [8]
Phylogenetic distinctiveness *	Amount of unique evolutionary history that species harbors [9]
Extinction rate	Ratio of vertebrate extinctions to the endemic fauna occurred within island [3]

† the average value was calculated for each island to obtain a unique value per island
* the average value was calculated for mammal species occurring on each island to obtain a unique value per island

Supplementary Methods – Procedure employed to select protected areas

We collected spatial information on Protected Areas (PAs) from the World Database on Protected Areas (WDPA) database available at: http://protectedplanet.net/[8]. The database was downloaded on 24th July 2018. The original database included several PAs designations not object to our study, such as areas for the protection of marine habitats/species. We then applied a 3-steps procedure which allowed us to build a dataset on PAs distribution across the 340 islands, like did by Parravicini *et al.* (2014)[10].

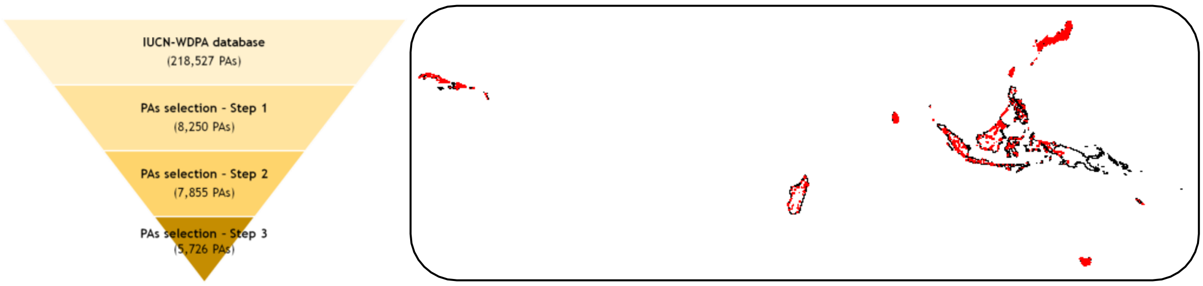

Supplementary Figure 12 - (left)Procedure employed to select protected areas across the 340 islands; (right) Location of the 5,729 PAs across the 340 islands

Step 1 - Download IUCN-WDPA database

The IUCN-WDPA database was intersected with the location of the 340 islands. 8,250 PAs covering a total surface of 8,589,083 km² were extracted.

Step 2 - Eliminating PAs with the goal of protecting habitat and species not object to the present study

A further filtering (including two steps) was needed because several PAs have been designated to protect habitats or species not considered in the present study (e.g. marine habitats, bird species).

First, we excluded PAs whose origin ("Marine" field) was recorded as 'marine'. The database included 8,111 PAs covering a total surface of 2,259,884 km².

In a second time, we evaluated the description of the "Designation" field in the original IUCN-WDPA database to remove PAs with irrelevant designations (i.e. Coastline Marine Resource Development Area, designated sea area, Common fishery right area, District Marine Protected Areas, Fish Sanctuary, Locally Managed Marine Area, Marine Conservation Area, Marine Mammal Sanctuary, Marine Managed Area, Marine National Park, Marine Nature Recreation Park, Marine Nature Reserve, Marine Park, Marine Park and Sanctuary, Marine Protected Area, Marine Reserve, Marine Sanctuary, Marine Wildlife Reserves, National Marine Reserve, Protected Seascape, Seagrass Sanctuary, Bird Sanctuary, Land acquired by Conservatoire du Littoral (national seaside and lakeside conservancy), National Estuarine Research Reserve, Protected Landscape/Seascape - Buffer Zone, Protected Natural Landscape/Seascape, Protected Water Surface). The database included 7,855 PAs covering a total surface of 2,178,836 km².

Step 3 - Eliminating MPAs belonging to IUCN categories V and VI

A further filtering was used in attempt to eliminate PAs that are not subject to strict protection measures. We thus eliminated MPAs belonging to IUCN categories V and VI, and also those with status indicating as 'not reported' and 'not applicable'. The database after removing meaningless designations included 5,726 PAs covering a total surface of 374,990km².

Supplementary References

1. Fick, S. E. & Hijmans, R. J. WorldClim 2: new 1-km spatial resolution climate surfaces for global land areas. Int. J. Climatol. **37**, 4302–4315 (2017).
2. Williams, J. W., Jackson, S. T. & Kutzbach, J. E. Projected distributions of novel and disappearing climates by 2100 AD. *Proc. Natl. Acad. Sci. U.S.A.* **104**, 5738–5742 (2007).
3. IUCN. The IUCN Red List of Threatened Species - version 2018-2. *http://www.iucnredlist.org* (2018).
4. Wilman, H. *et al.* EltonTraits 1.0: Species-level foraging attributes of the world's birds and mammals. *Ecology* **95**, 2027–2027 (2014).
5. Pacifici, M. *et al.* Generation length for mammals. *Nat. Conserv.* **5**, 89–94 (2013).
6. Leclerc, C., Villéger, S., Marino, C. & Bellard, C. Global changes threaten functional and taxonomic diversity of insular species worldwide. *Divers. Distrib.* **26**, 402–414 (2020).
7. Weigelt, P., Jetz, W. & Kreft, H. Bioclimatic and physical characterization of the world's islands. *Proc. Natl. Acad. Sci. U.S.A.* **110**, 15307–11532 (2013).
8. UNEP-WCMC and IUCN. Protected Planet: The World Database on Protected Areas (WDPA), Cambridge, UK: UNEP-WCMC and IUCN. *www.protectedplanet.net* (2018).
9. Faurby, S. *et al.* PHYLACINE 1.2: The Phylogenetic Atlas of Mammal Macroecology. *Ecology* **99**, 2626–2626 (2018).
10. Parravicini, V. *et al.* Global mismatch between species richness and vulnerability of reef fish assemblages. *Ecol. Lett.* **17**, 1101–1110 (2014).
