# Supplementary material for: Future climate change vulnerability of endemic island mammals
Source: Nat Commun. 2020 Oct 2;11:4943. doi: 10.1038/s41467-020-18740-x (PMC7532204; doi:10.1038/s41467-020-18740-x)
Supplement: Supplementary file 3 — Reporting Summary [file 41467_2020_18740_MOESM3_ESM.pdf]

nature research

Corresponding author(s): Camille LECLERC

Last updated by author(s): Jun 24, 2020

Reporting Summary

Nature Research wishes to improve the reproducibility of the work that we publish. This form provides structure for consistency and transparency in reporting. For further information on Nature Research policies, see Authors & Referees and the Editorial Policy Checklist.

Statistics

For all statistical analyses, confirm that the following items are present in the figure legend, table legend, main text, or Methods section.

n/a	Confirmed	
☐	☒	The exact sample size (*n*) for each experimental group/condition, given as a discrete number and unit of measurement
☐	☐	A statement on whether measurements were taken from distinct samples or whether the same sample was measured repeatedly
☐	☒	The statistical test(s) used AND whether they are one- or two-sided<br>*Only common tests should be described solely by name; describe more complex techniques in the Methods section.*
☐	☒	A description of all covariates tested
☐	☒	A description of any assumptions or corrections, such as tests of normality and adjustment for multiple comparisons
☐	☒	A full description of the statistical parameters including central tendency (e.g. means) or other basic estimates (e.g. regression coefficient) AND variation (e.g. standard deviation) or associated estimates of uncertainty (e.g. confidence intervals)
☐	☒	For null hypothesis testing, the test statistic (e.g. *F*, *t*, *r*) with confidence intervals, effect sizes, degrees of freedom and *P* value noted<br>*Give P values as exact values whenever suitable.*
☐	☐	For Bayesian analysis, information on the choice of priors and Markov chain Monte Carlo settings
☐	☐	For hierarchical and complex designs, identification of the appropriate level for tests and full reporting of outcomes
☐	☒	Estimates of effect sizes (e.g. Cohen's *d*, Pearson's *r*), indicating how they were calculated

Our web collection on statistics for biologists contains articles on many of the points above.

Software and code

Policy information about availability of computer code

Data collection	No software was used to collect data.
Data analysis	All data analysis was performed in R Version 3.6.0.

For manuscripts utilizing custom algorithms or software that are central to the research but not yet described in published literature, software must be made available to editors/reviewers. We strongly encourage code deposition in a community repository (e.g. GitHub). See the Nature Research guidelines for submitting code & software for further information.

Data

Policy information about availability of data

All manuscripts must include a data availability statement. This statement should provide the following information, where applicable:

- Accession codes, unique identifiers, or web links for publicly available datasets
- A list of figures that have associated raw data
- A description of any restrictions on data availability

The climate change vulnerability data generated and analysed during this study are included in the Source Data file. The other datasets used in this study are derived from published sources, cited in the data availability section, the methods section and summarized in Table S2.

Field-specific reporting

Please select the one below that is the best fit for your research. If you are not sure, read the appropriate sections before making your selection.

☐ Life sciences ☐ Behavioural & social sciences ☒ Ecological, evolutionary & environmental sciences

Ecological, evolutionary & environmental sciences study design

All studies must disclose on these points even when the disclosure is negative.

Study description	Here we assess the future climate change vulnerability of islands, by using data on the distribution of endemic terrestrial mammals, available from the IUCN, and on islands, available from Weigelt et al. (https://doi.org/10.1073/pnas.1306309110). Specifically, we calculate three components (exposure, sensitivity, and adaptive capacity) through collate data on islands environmental conditions and on the ecology of species (including climate, species specialization, and geographic isolation) from a range of published sources. We then combine and adapt trait-based and quantitative vulnerability frameworks, to assess the vulnerability of islands to climate change by 2050.
Research sample	We used data on the distribution of 873 endemic terrestrial mammals species currently available and acquired from the IUCN (version 2018, https://www.iucnredlist.org). By overlapping mammal occurrences with islands spatial data from Weigelt et al. (https://doi.org/10.1073/pnas.1306309110), 340 islands from 14 archipelagos were considered.
Sampling strategy	For this study we used islands where endemic mammals occurred and that are represented by at least 10 grid points of climate data. This provided an overall sample size of 340 islands and .873 endemic terrestrial mammals. In addition, to characterise vulnerability components and variables, eight datasets were used. These datasets were chosen owing to their free access and their taxonomic or spatial coverage. These datasets cover a large number of biological and environnemental variables generally used during climate change vulnerability assessment.
Data collection	All of the datasets used in this study are from published sources, cited in the methods. CL collected and recorded text or excel files and also spatial files (i.e. shapefiles and raster) from the different public sources. Particularly, current and future global climate data are available at http://www.worldclim.com/version2. Data about island geographic isolation and spatial distribution are available at https://datadryad.org/stash/dataset/doi:10.5061/dryad.fv94v. Protected areas data are available at http://protectedplanet.net/. Species phylogeny is available at https://megapast2future.github.io/PHYLACINE_1.2/. Species generation length are available at https://datadryad.org/stash/dataset/doi:10.5061/dryad.gd0m3. Data about species habitat, extinction, and spatial distribution are available at https://www.iucnredlist.org/. Species diet data are available at http://www.esapubs.org/archive/ecol/E095/178/metadata.php. The two last data sources were also used to compute ecological redundancy.
Timing and spatial scale	Data on species' distributions were acquired from the IUCN in August 2018. Variables characterizing the three vulnerability components (exposure, adaptive capacity, and sensitivity) come from different published sources in the 2010's. Climate data were acquired from Worldclim for two periods: current period that is an averaging of climate data from 1970–2000 and future projections for 2041–2060 ("2050"). All data were acquired at a global scale and restricted and analyzed at the scale of the 340 islands and 873 endemic terrestrial mammals.
Data exclusions	We excluded islands represented by less than 10 climate data points that provide an insufficient climate change information to assess vulnerability.
Reproducibility	To ensure that our results were robust we applied three types of transformations of the values of vulnerability and associated components, and tested their correlation. Reproducibility was successful.
Randomization	Initially, the analysis was performed using all available data for the 340 islands where occurred the endemic terrestrial mammals. Species were organized by islands where they occur. Analyses were first performed at a island scale, but then split into 14 archipelagos as defined by Weigelt et al. (https://doi.org/10.1073/pnas.1306309110).
Blinding	Blinding was not relevant for our study as we only used data from published sources and made no specific a priori hypotheses.

Did the study involve field work? ☐ Yes ☒ No

Reporting for specific materials, systems and methods

We require information from authors about some types of materials, experimental systems and methods used in many studies. Here, indicate whether each material, system or method listed is relevant to your study. If you are not sure if a list item applies to your research, read the appropriate section before selecting a response.

Materials & experimental systems

n/a	Involved in the study
☒	☐ Antibodies
☒	☐ Eukaryotic cell lines
☒	☐ Palaeontology
☒	☐ Animals and other organisms
☒	☐ Human research participants
☒	☐ Clinical data

Methods

n/a	Involved in the study
☒	☐ ChIP-seq
☒	☐ Flow cytometry
☒	☐ MRI-based neuroimaging
